# Ergodic Sets as Cell Phenotype of Budding Yeast Cell Cycle

**DOI:** 10.1371/journal.pone.0045780

**Published:** 2012-10-01

**Authors:** Robert G. Todd, Tomáš Helikar

**Affiliations:** Department of Mathematics, University of Nebraska at Omaha, Omaha, Nebraska, United States of America; Virginia Tech, United States of America

## Abstract

It has been suggested that irreducible sets of states in Probabilistic Boolean Networks correspond to cellular phenotype. In this study, we identify such sets of states for each phase of the budding yeast cell cycle. We find that these “ergodic sets” underly the cyclin activity levels during each phase of the cell cycle. Our results compare to the observations made in several laboratory experiments as well as the results of differential equation models. Dynamical studies of this model: (*i*) indicate that under stochastic external signals the continuous oscillating waves of cyclin activity and the opposing waves of CKIs emerge from the logic of a Boolean-based regulatory network without the need for specific biochemical/kinetic parameters; (*ii*) suggest that the yeast cell cycle network is robust to the varying behavior of cell size (e.g., cell division under nitrogen deprived conditions); (*iii*) suggest the irreversibility of the *Start* signal is a function of logic of the G1 regulon, and changing the structure of the regulatory network can render start reversible.

## Introduction

Complex network structures can be found across the biological spectrum, and growing evidence indicates that these biochemical networks have evolved to perform complex information processing tasks in order for the cells to appropriately respond to the often noisy and contradictory environmental cues [1]. While reductionist techniques focus on the local interactions of biological components, the systems approach aims at studying properties of biological processes as a result of all components and their local interactions working together [2].

A wide spectrum of modeling techniques ranging from continuous frameworks utilizing differential equations to discrete (e.g., Boolean) techniques based on qualitative biological relationships exist [3]–[5]. Each modeling technique is based on different assumptions and hence comes with different advantages and disadvantages. Differential equation models can depict the dynamics of biological systems in great detail, but depend on a large number of difficult-to-obtain biological (kinetic) parameters. On the other hand, discrete modeling frameworks, namely Boolean networks, are qualitative and parameter-free, which makes them more suitable to study the dynamics of large-scale systems for which these parameters are not available. Furthermore, probabilistic Boolean networks (PBN) enhance the discrete framework by allowing for uncertainty and stochasticity (e.g., [6], [7]).

It has been proposed that the irreducible sets of states (i.e., ergodic sets) of the corresponding Markov chain in probabilistic Boolean network models (PBNs) are the stochastic analogue of the limit cycle in a standard Boolean network, and should thus represent cellular phenotype [8]. However, often PBNs with perturbations are studied to include internal noise, rendering the search for the irreducible sets trivial (as the whole state space constitutes a single irreducible component). Furthermore, this makes the determination of the limiting distribution of the corresponding Markov chain and the interpretation of those results in light of the biology challenging even for moderately sized models [9]–[11].

Using the idea from [1] to introduce stochasticity to Boolean models via control nodes, herein we determine and examine the nature of ergodic sets of a regulatory network governing each phase of the cell cycle of budding yeast, *Saccharomyces cerevisiae*. The budding yeast cell cycle has been modeled previously using Boolean approaches (e.g., [5], [12], [13]) and probabilistic Boolean approaches (e.g., [7], [14], [15]). We expand on previous works by considering each phase of the cell cycle as an individual evolving system. The logic of the model used in this study was developed from the description of the yeast cell cycle interactions given in [12]. Using this model we show that as suggested in [8], irreducible sets of states can correspond to cellular phenotype. This approach enables us to model and visualize richer dynamical properties of each phase and the cell cycle as a whole. In particular, we show that under stochastic external signals the continuous oscillating waves of cyclin activity and the opposing waves of CKIs that form the cell cycle engine can emerge from the logic of a relatively simple regulatory network without the need for specific biochemical/kinetic parameters. Furthermore, by considering each phase of the cell cycle as an individual system represented by an “ergodic set”, we are able to more directly and precisely compare the model dynamics with experimental studies. Specifically, results of [16] as interpreted graphically at cyclebase.org reveal relatively precise similarities. We also observe good agreement between our oscillating cyclin activities and recently published analyses of cyclin activities using fluorescent microscopy in [17]. The improved approach to the modeling of the yeast cell cycle enables us to visualize other qualitative features of the system: the secondary activation of a number of G1-cyclins later in the cell cycle [16] and the renewed reversibility of *Start* upon the removal of the Cln2-SBF-Whi5 feedback loop [18]. We also capture the same robustness to internal perturbations as described in [5], however we extend this result and conclude that each phase of the yeast cell cycle (and thus as the cell cycle as a whole) is robust in the face of the variable behavior of the cell size. Within the model, this results in the post-Start commitment to the cell cycle and the ability to complete a single round of division under deprived nutrition conditions [19].

## Results

### Modeling the Cell Cycle

**Figure 1:**
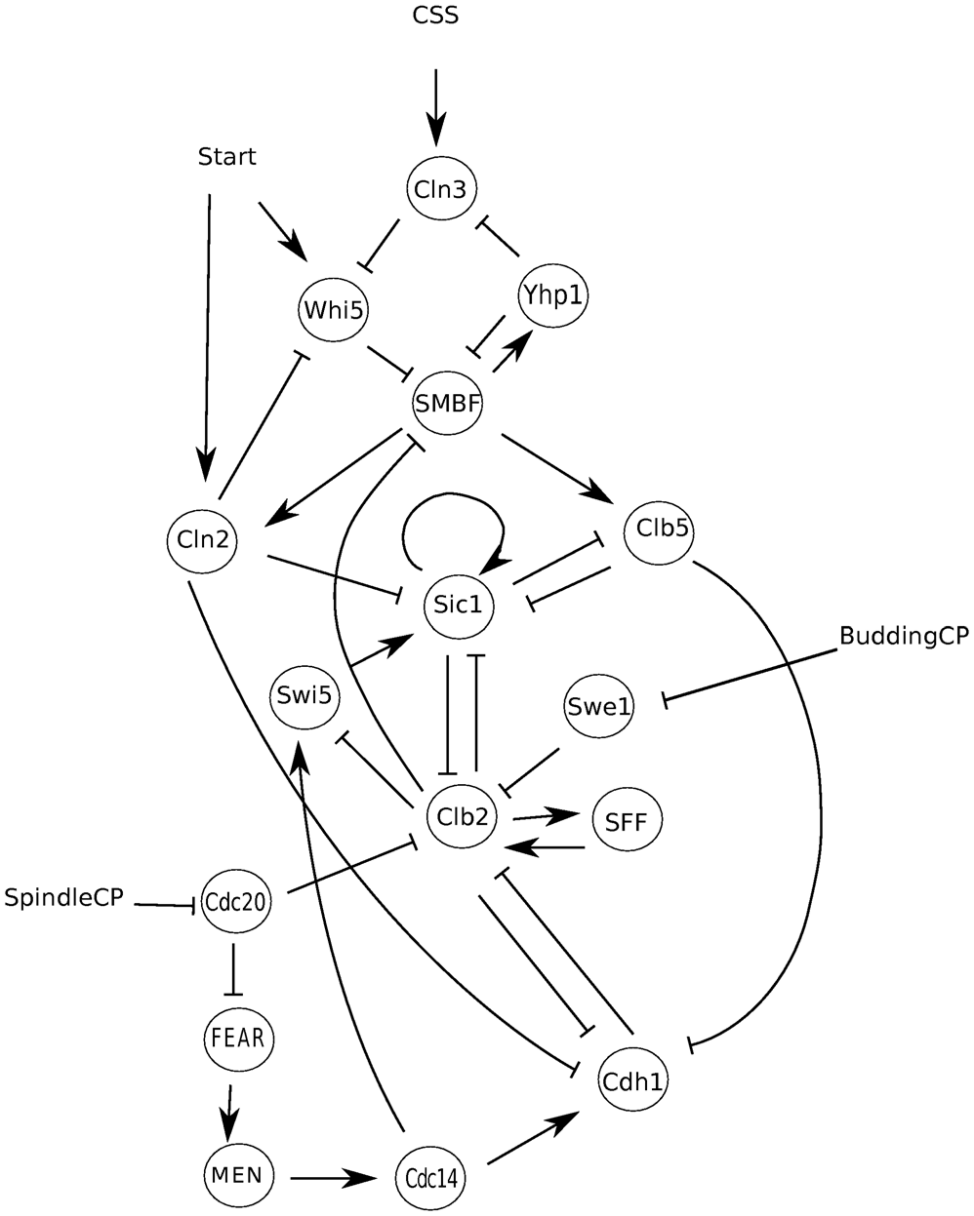
Regulatory Graph for Budding Yeast.

The budding yeast cell cycle involves hundreds of species and interactions [20]. In order to keep the mathematical analyses manageable, we consider a much smaller network consisting of some key players (see *Methods* for a narrative description of the cell cycle). The logic of our network was constructed based on the descriptions of the cell cycle interaction as given in section 3.1 of [12], which is an expansion of the network found in [5]. All nodes, the species they represent and the logic associated to each node, are available in The Cell Collective (www.thecellcollective.org). Figure 1 shows the static interaction graph of the model. The model used in this study has four external inputs: cell size signal (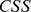) to model cell growth, the Start checkpoint (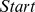), the budding (or morphogenic) checkpoint (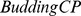) and the spindle assembly checkpoint (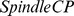). Each of these external inputs plays a different role. First consider 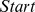, 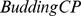, and 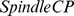. Each external input is incorporated into the logic of an internal node(s) so as to mimic the biological behavior of a checkpoint. Activating one of these external inputs (setting it to 1) indicates that the corresponding checkpoint has been satisfied. In pre-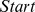 cells, 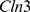 cannot inhibit 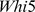 nor can 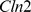 be activated unless the critical size threshold has been reached; hence 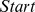 is integrated into the logic of Whi5 and Cln2 as follows: if 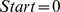 then 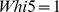 and 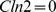 [21]–[24]. The external input 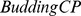 corresponds to the correct formation of the bud neck and the localization and subsequent degradation of Swe1 [25]–[27]. As such, we say that if 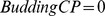 then 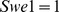. Lastly, 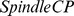 corresponds to the spindle assembly checkpoint which modulates the activation of Cdc20: if 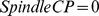 then 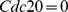 [28], [29].

Finally, 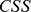 is a signal representing cell size. It is known that cell size regulates the cell cycle via its correlation with Cln3 levels. The mechanism governing this regulation involves a complex network of biochemical interactions [30], [31], and has been omitted for simplicity. Unlike the external nodes representing cell cycle checkpoints, which are binary in nature (either satisfied or not), the 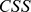 external input is inherently continuous (cell size varies continuously over time). To represent this continuous signal passed from the cell and its environment to activate 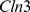 at a given moment, a probability (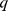) that 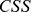 is active is defined: 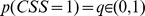. This signal is relative as 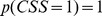 indicates 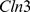 is receiving the strongest activation signal.

The cell cycle was modeled as a sequential activation of the checkpoints. In other words, the pre-start or G1 phase was modeled by setting all checkpoints to 0. The G1/S phase is modeled by setting 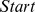 to 1. For the G2/M phase, 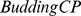 is set to 1; finally, the M/G1 phase is modeled via the activation of the 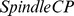 checkpoint. Hence, the cell cycle as a whole results in a sequence of *probabilistic Boolean control networks (PBCNs)* (see the *Methods* section for detailed discussion of PBCNs) as follows:
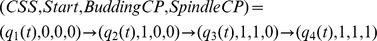



Call these *PBCN1*, *PBCN2*, *PBCN3*, *PBCN4*, where each 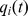 is the control function governing CSS during each modeled phase. The question is then: does each *PBCN* behave in accordance with the phase assigned to it by the status of its checkpoints? In the next section, the results of our analyses of the dynamics of these PBCNs are presented.

### Cyclin Activity Profiles Correspond to Ergodic Sets

**Figure 2:**
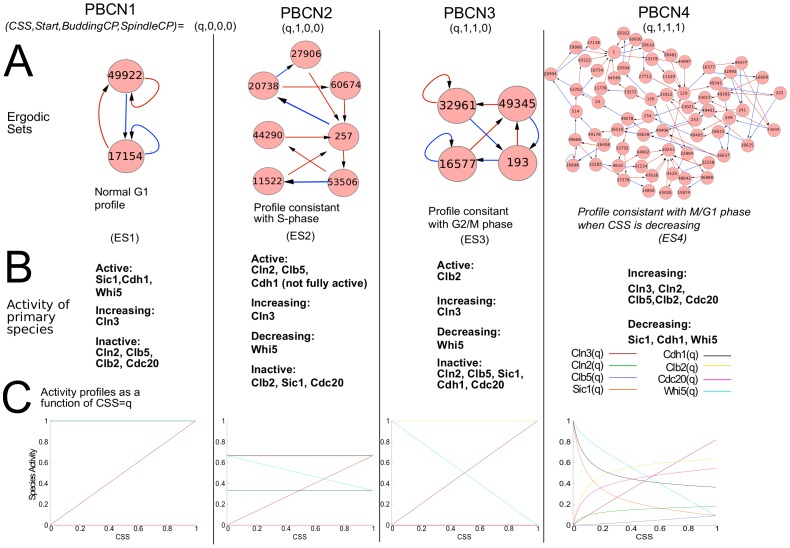
Analysis of each PBCN. A) Ergodic sets (consisting of network states) for the individual *PBCN*s corresponding to individual cell cycle phases. Each *PBCN* was constructed by changing the combination of satisfied checkpoints; the “activity” of the Cell Size Signal (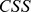) external node is defined by a probability 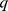. Ergodic sets are visualized as nodes corresponding to network states (represented by their binary number +1) connected by arrows illustrating the flow of these states. Red arrows correspond to 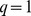, blue arrows correspond to 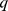 = 0. Discussion of the individual ergodic sets and their biological meaning can be found in the main text. B) Activity profiles (“signatures”) of cyclins during each modeled cell cycle phase. C) Plots of cyclin activity functions as found by computing stationary distribution analytically using Maple 15, as discussed in the main text.

To demonstrate how PBCNs can be used to visualize and analyze the dynamics of biological systems, we first show that ergodic sets correspond to cell phenotypes; i.e., cyclin activity patterns of the individual cell cycle phases, in our case. Each *PBCN* was analyzed, and ergodic sets were calculated. The question we then asked: Do the cyclin activity functions of the individual ergodic sets (and hence the modeled cell cycle phases) correspond to the cyclin activity profiles during the cell cycle as seen in the laboratory? In other words, does our model represent the biological reality? In fact, the results of our analyses (discussed below) indicate that the presented model accurately captures many of the features of the species’ expected behavior (i.e., their activity levels) during each cell cycle phase. In Figure 2 the ergodic sets associated with each *PBCN* and the corresponding activity functions of key cyclins as a function of 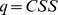 are summarized. For each of the *PBCNs* exactly one ergodic set was found (ES1-4, Figure 2A). For (ES1) the cyclin activity functions (column 1 in Panel C) is consistent with pre-start G1 cells. The cyclin activity functions of ergodic sets for *PBCN2* and *PBCN3* are consistent with the G1/S and G2/M phase of the cell cycle, respectively (columns 2 and 3 in Figure 2A and B). Finally, during the last stage, the cyclin activity functions of *PBCN4* is consistent with M/G1 phase when 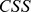 is decreasing. That is, the cyclin dependent kinases (e.g., 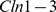, 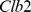, etc.) deactivate while the cyclin kinase inhibitors (e.g., 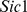 and 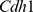) reactivate. Thus we see that in fact each PBCN does behave according the phase assigned to it by the status of its checkpoints.

In order to model the dynamics of the cell cycle as a whole we must consider how 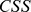 is changing over time. Choosing an appropriate 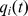 for 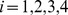 as the control functions for 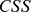 for each corresponding phase we may see the behavior of each species across the cell cycle as a whole. As time is arbitrary in our model, we chose 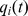 to have the 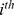 quarter of the unit interval as its domain, and thus the modeled cell cycle to take one unit of time. To organize the transition from one phase to the next we suppose that if one concatenates those functions into a single function 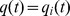 if 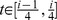 the overall behavior of 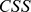 should mimic cell size; i.e., it should grow for the majority of the cell cycle and drop at the end. Furthermore, we assume that Cln3 peaks when it is receiving the maximum signal. It has been shown that the level of Cln3 rises and falls over the cell cycle and peaks sometime during M phase [16], [32]. Thus we let 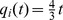 with domain 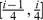 for 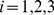 and 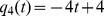 with domain 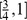. (Note that the dynamical properties of the model are highly robust to variations of the function, and thus our choice of the control functions, as discussed in the *Robustness* section. ).

**Figure 3:**
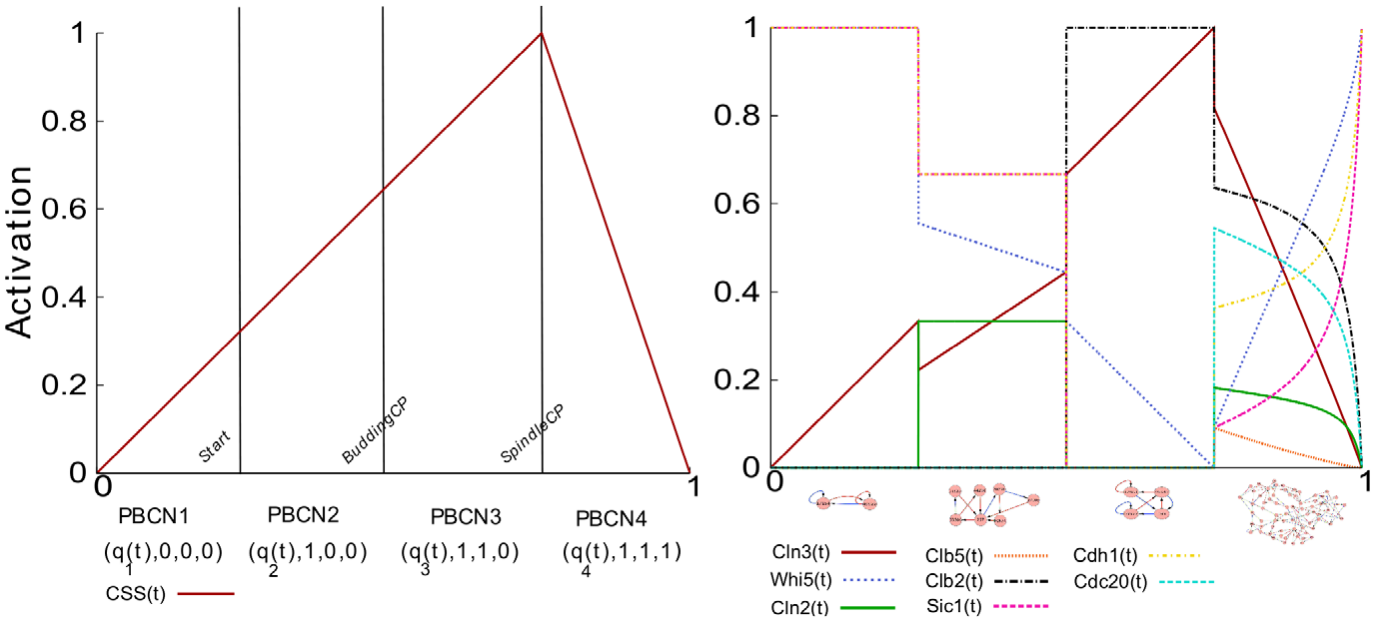
The cell cycle in relative time. The left hand side depicts the control function for 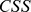 along with the points in time where checkpoints are activated. The right hand side depicts the concatenated activity profiles of the corresponding ergodic sets composed with 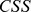 control function. All species appear during each phase, though several my take on the same value, including 0.

**Figure 4:**
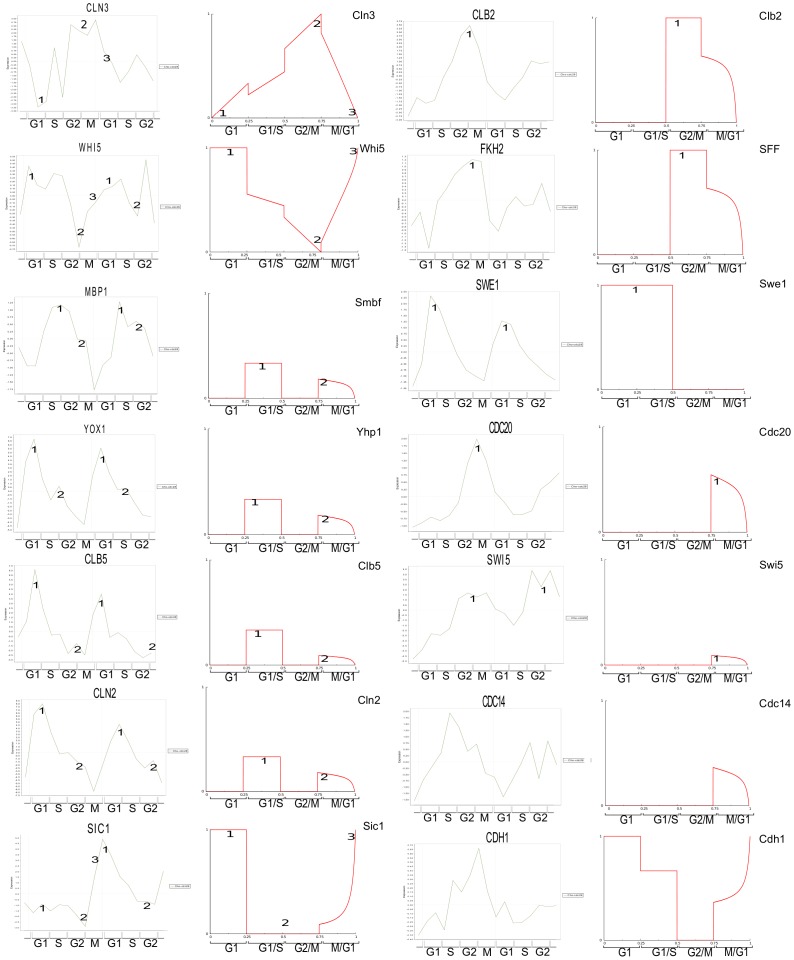
Comparison between our analytically calculated results (red) and the experimental results in [**16**] (green) via cyclebase.org. Each node in the network may represent several species. In the case that a node represents more than one species its calculated activity profile is compared to the experimental activation of the species to which it most clearly correlates. For example, the node Yhp1 in our model represents the species YHP1 and YOX1. We thus compare the calculated profile of the node Yhp1 to YOX1, as they appear to have the best correspondence. Numbered peaks and valleys identify our interpretation of the correlation between plots. The species corresponding to each node can be found at thecellcollective.org.

Consequently for each species, a piecewise function that governs its activity across the cell cycle was constructed by composing each node’s activity function with 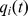 during each corresponding phase of the cell cycle. In Figure 3, one can see the control function for 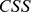 over the whole cell cycle on the left and the corresponding activity of selected cyclins across the whole cell cycle on the right. It is clear that we are able to reproduce the general structure of the cell cycle. Cyclin kinase inhibitors are active in G1, followed by their deactivation and the activation of the G1 cyclins Clb5 and Cln2. Then the G1 cyclins deactivate and Clb2 activates. Finally Clb2 deactivation correlates with Cdc20 activation as the cell progresses through M phase, and the reactivation of the CKIs. (See *Methods* for a narrative description of the cell cycle.) Direct comparison of the shape of the calculated activity profiles to experimental studies in [16] (via cyclebase.org) revealed a strong correspondence (Figure 4). Exceptions to this correspondence with results from [16] were the dynamics of Cdc14 and Cdh1. Our model predicts that Cdc14 is activated late in the M phase (while inactive during the previous phases). This behavior appears however to be consistent with another study that suggested that Cdc14 activation occurs in late mitosis [33]. Also, while the activation profile of Cdh1 predicted by our model doesn’t agree with the results in [16], it appears to be consistent with the activation profile described in Figure 2 in [4] (as well as all other species common to each model). Thus not only does our model’s results compare to laboratory results but also to the results of a differential equation model. Note that the activity levels of 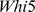, 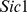, 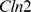, 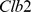, and 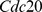 also qualitatively correspond to the combination of activity and localization measured in the cell (see Figure 3 in [17]). Together, these data suggest that, in fact, ergodic sets can model cell phenotypes. Furthermore, as visualized in Figure 3 and Figure 4, a secondary peak of the G1 Cyclins (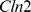 and 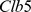 in particular) was found as the cell transitions from *PBCN3* to *PBCN4*. In fact, this phenomenon was also observed in the laboratory [16]. This is also a feature of the cyclin activity profiles of the respective ergodic sets. Notice also that this peak is not purely a result of the function that we chose for 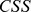. While the shape of the peak may change, its existence is intrinsic to the logic of the network; that is, so long as 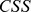 does not drop instantly to zero when the spindle assembly checkpoint has been satisfied, there will be some sharp rise and fall of 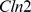 and 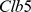.

### Start, Irreversibility, and Commitment to the Cell Cycle

The irreversible nature of the *Start* signal was explored in [18] by investigating the positive feedback loop that exists between 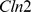, 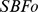 (“
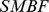
” in our model) and 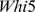 (see Figure 1). As can be seen in Figure 3B, the bi-stability of 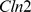 is clearly represented in the transition from the G1 ergodic set (ES1) where 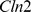 is inactive to the S-phase ergodic set (ES2) where 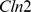 is active. Notice that in *PBCN1*, the 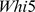-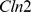-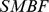 feedback loop cannot be initiated, as 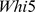 is active and 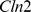 is inactive until the 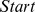 signal has been received. On the other hand, in the post-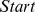 phase (i.e., *PBCN2*), 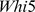 is now inhibiting itself as a result of the 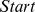-activated feedback loop. This suggests that the feedback loop is inherent to the irreversibility of 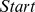.

**Figure 5:**
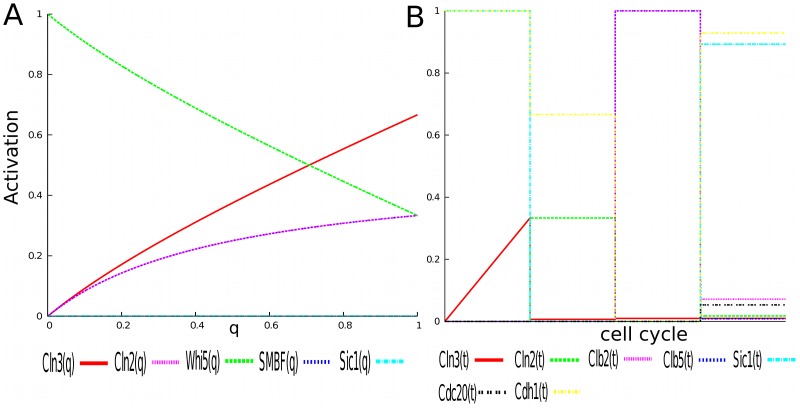
Irreversibility and commitment to cell cycle. A) Cln2 becomes inactive and Whi5 reactivates when 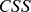 stimulation is removed. Thus breaking Whi5-SMBF-Cln2 feedback loop makes Start reversible. B) Modeled cell cycle under nitrogen deprivation. 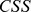 is linear during the G1 phase and drops to.01 there after.

In the aforementioned work [18], the authors showed that removing 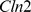 from the feedback loop allows the reactivation of 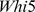 following an exogenous pulse of 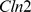, rendering Start reversible. To perform an analogous inquiry on our model, and to investigate the role of the feedback loop, we eliminated 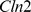 as an upstream regulator of 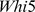 and re-analyzed *PBCN2*. A single ergodic set was found, whose cyclin activity profile is pictured in Figure 5A. The functions from the activity profiles of ES1 and ES2, that govern 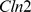 when the feedback loop is present are constant functions, and thus have no dependence on 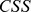. In contrast, 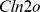 and 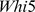 activities are now a function of 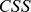 (Figure 5A). In other words, if the 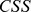 stimulus is removed from 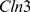, 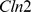 becomes inactive and 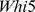 reactivates, indicating a return to G1 phase and a renewed possibility of G1 arrest due to mating pheromone [34]. The transition to S phase is now reversible. Though the context of our model and what was done in [18] are different, the result is the same – the irreversibility of the G1/S transition is dependent on the positive feedback loop.

**Figure 6:**
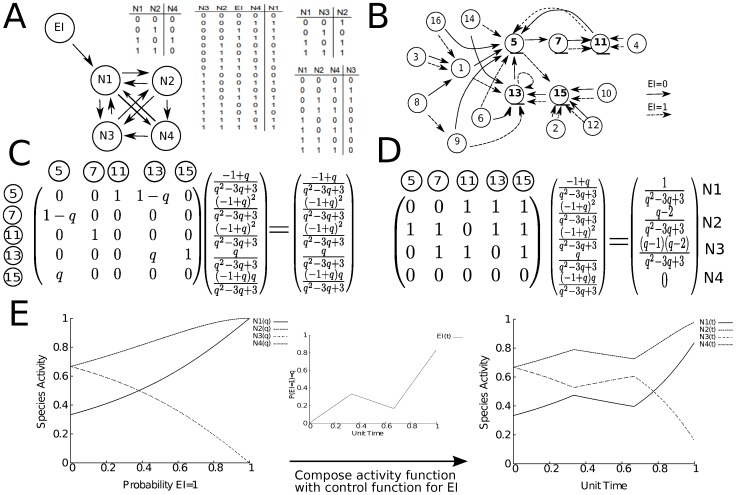
An example calculation. A) A diagram of a sample network with one external input. The logic of the internal nodes is represented with Boolean truth tables. B) The state space associated with the network. Nodes are labeled by 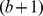 where 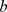 is the binary number corresponding to the activity of (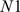,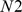,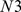,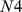). Supposing that the probability that 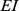 is active is 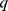, the state space is traversed on dashed arrows with probability 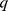 and solid arrows with probability 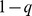. Nodes labeled with an underline constitute the ergodic set. C) On the left is the probabilistic transition matrix that governs the system once it has reached the ergodic set. With the matrix is associated a unit modulus eigenvector that provides the invariant distribution for the system. D) Each state of the ergodic set gives the activities of the internal nodes. Taking the sum of these binary vectors weighted by the invariant distribution gives the likelihood that a particular node is active. Thus on the right of this expression is the activity function of each node in the ergodic set. Note that the activity function is continuous for 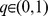. E) In the left graph, the activity function of each node is plotted as a function of 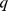. In the middle graph, an arbitrary function for 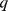 (or the activity level of 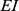) is plotted as a function of time. In the graph on the right side, the activities of the network nodes is plotted as a function of the composite function for 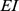 in time (as designed in the middle graph).

That the functions for the cyclins in the G1, S, and G2 phases are constant has another implication for our model. Specifically, once the 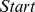 signal has been received, the typical (oscillating) activity profiles of the key cyclins will ensue even when stimulus of Cln3 by cell size is incoherent, so long as the checkpoints are satisfied. In other words, once the cell receives the 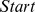 signal, it commits to a round of cell division.

### Robustness

Robustness of biological systems is critical to the proper function of processes such as the cell cycle. Within our modeling regime noise is interpreted as the systems’ sensitivity to the control function, and the robustness of the ergodic sets to random perturbations, respectively. To consider the system’s sensitivity to the control function, we considered the activity functions of the ergodic sets. As noted in the previous section, the activity functions governing most of the key species in the system are constant, and hence independent of 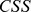 during the first three phases of the cell cycle. In particular, 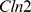, 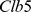, and 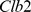, which drive bud formation, DNA replication, and mitosis, respectively, have their activities governed by constant functions. This indicates that so long as the checkpoints are appropriately activated (i.e., the environment is stable enough for the successful completion of the current phase), the modeled cell will progress through the cell cycle independently of 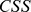 (i.e., cell size). Therefore our model is robust to the variable behavior of the cell growth.

Furthermore, this is also consistent with the findings in [19] that a cell deprived of nitrogen will proceed through one round of division and arrest in G1. We modeled this scenario by removing the cell size signal (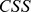) right after *Start* has been satisfied. Consistent with [35], as a control function we chose 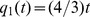 for 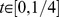 and 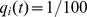 for 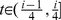, 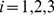. The dynamics of modeled cyclins are depicted in Figure 5B. During the first three phases, the activities of the species are the same as the normal cell cycle (Figure 2). The activities of the species during the last phase are also consistent with a cell in the G1 phase. That is, the modeled cell has completed a round of division and arrested in G1. This may suggest that the phenomenon of completing a cell cycle without appreciable growth is a consequence of the robustness of the cell cycle to variable external environments, and is inherent to the logic of the biological regulatory network governing the yeast cell cycle.

In addition to being able to represent cellular phenotypes, the calculated ergodic sets (and the number thereof) in the previous section have another implication. Similar to attractors in Boolean network, ergodic sets can provide insights into the robustness of the modeled biological systems.

A standard approach to analyze robustness is to consider the basins of attractions of each attractor and interpret its relative size as a measure of stability (e.g., [5]). The concept of a basin of attraction for an ergodic set in a *PBCN* is not well defined; this is due to the fact that a random walk initiated from a single state in the state space may arrive at different ergodic sets. However, each of the *PBCNs* have a single ergodic set which means that any perturbation will eventually return to the ergodic set (and can be modeled by its associated cyclin activity functions). As such, we see that the modeled G1, G1/S, G2/M and M/G1 phases of the cell cycle are highly robust in the face of perturbations. Together, our results suggest that each phase of the modeled cell cycle is robust as well as the cell cycle as a whole.

## Discussion

Results presented herein are twofold. First, as suggested in [8], we show that it is possible to model cellular phenotype as ergodic sets in the context of probabilistic Boolean control networks. In contrast to previous works utilizing Boolean models, our approach centers around understanding not only the cell cycle as a whole, but also its individual phases. Specifically, we modeled the cell cycle as a sequence of models, each representing an individual phase in the cycle. This approach has significant implications as to how the dynamics of the modeled cell cycle are interpreted and are compared with experimental studies. Specifically, in previous works the yeast cell cycle was modeled as a single system where the phases were represented as transient states leading to a (fixed point) attractor corresponding to the G1 phase [5], or as consecutive states in a cyclic attractor [12], [13]. Considering each phase as an evolving system of its own enabled us to capture continuous dynamics of key species during each phase and compare them to laboratory studies. Modeling each phase separately and transitioning between models via the activation of checkpoints is also consistent with the biological observations that it is not only kinase activity that causes phase transitions, but the completion of each phases task [36].

Similar to [5], [12], [13] we find that each phase of the cell cycle and thus the cell cycle as a whole is robust as measured by basin size, i.e. the existence of a single ergodic set for each phase. Stability of the yeast cell cycle has also been considered in the framework of probabilistic Boolean networks, concluding the cell cycle attractor is robust to internal noise [7], [15], [37]. However, this approach is incompatible with our goal of exploring the relevance of ergodic sets as it renders the entire state space a single ergodic set. Modeling extracellular signals as continuous variables (i.e., cell size) allowed us show the stability of the yeast cell cycle network under different choices of control functions, a question precluded by previous modeling techniques. Lastly, taking the perspective of qualitative activity introduced in [1] we are able to directly incorporate the role of cell size into our model. The further correlation of cell size with time also allows us to escape the discrete time of other Boolean network models.

Evidence is increasing that biological processes possess complex properties that emerge from the dynamics of the system working as a whole (e.g., [1], [30], [38]–[41]). To better understand these emergent properties, large-scale computational models of the complex biological interactions will be needed. The size of the budding yeast cell cycle network in this work is relatively small and makes the analytic calculations manageable. Larger and more comprehensive models will be key in systems biology. For example, understanding how the cell controls checkpoints via additional regulatory network pathways, and how to incorporate this understanding into current models is of paramount importance. Thus the question of how to approach large networks is important in extending these results to truly life-size scales. To deal with such scales simulation techniques and software (such as The Cell Collective; http://www.thecellcollective.org) will be an important part of extending these results to large models.

## Methods

### Budding Yeast Cell Cycle

Newborn cells begin in the G1 phase of the cell cycle, where they start growing. It isn’t until the cell reaches a critical size that a round of division begins [42]. This transition point is referred to as *Start*, and is irreversible; that is, once the *Start* signal is received, the cell is no longer susceptible to G1 arrest due to mating pheromone, and the cell has committed to a round of division, [18], [42], [43]. The activity profile of the biochemical network underlying the cell cycle during the initial G1 phase is characterized by the increasing activity of the Cln3 cyclin in response to the cell’s increasing size, and the activity of the cyclin kinase inhibitor (CKI) Sic1 [42]. The transition to S phase occurs once the critical size has been reached, i.e. *Start* has occurred, and Cln1, 2 has become active and Sic1 has been inactivated. The inactivity of Sic1 allows the activation of Clb5. Having transitioned to S phase, the cells characteristic cyclin activity pattern is the activity of Cln1, 2 and Clb5 and the inactivity of Sic1. During S phase, Cln1, 2 allow bud and spindle-pole body formation, while the activity of Clb5 allows DNA replication [18], [23]. In G2 phase, Clb2 (the primary mitotic cyclin) accumulates [44], and Swe1 is degraded in the newly formed bud neck [25]–[27]. In fact, bud formation (along with other nuclear events) constitutes another quality control point: a morphogenic checkpoint [27]. The activity of Clb2 is sustained into early M phase [44], [45]. Thus one may say that active Clb2 (and inactive Sic1) characterizes the G2/M phase of the cell cycle. Further progression through M phase is governed by another checkpoint: the spindle assembly checkpoint. Once the chromosomes are correctly aligned on the mitotic spindle, Cdc20, a co-factor of the ubiquitin ligase anaphase-promoting complex/cyclosome (APC/C), is released from inhibition. The cell then will progress through the rest of M phase and divide into a mother and daughter cell in G1 phase, awaiting another round of division. Thus, the activation and deactivation of Cdc20 and the corresponding recovery of the Sic1 and Cdh1 and characterize the M/G1 phase of the cell cycle [29], [46]. It is this oscillating activity of cyclin-dependent kinases that “act as the master regulator for cell cycle progression” [47].

The complete model is freely available for download and further modifications in The Cell Collective software at http://www.thecellcollective.org; [48].

### Modeling Framework

As noted in the *Introduction* section, the modeling framework herein was suggested by [1]. The essential perspective of this framework is to suppose that at every moment of time, our biological system is being modeled by the stationary distribution of an irreducible Markov chain, whose states are an irreducible subset of the state space for a probabilistic Boolean network (which itself is a *reducible* Markov chain).

Consider a collection of n nodes 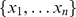, representing biological entities, each taking a value in 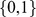, and 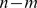 Boolean functions 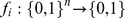, 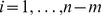, where the function 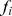 is the logical rule governing 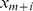. Call the nodes 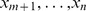 internal nodes and call nodes 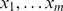 external inputs, as they are not governed by a Boolean function. Decompose the state space of the original 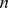 nodes as the direct sum 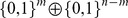 so that for 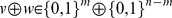 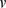 represents the state of the external inputs and 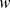 represents the state of the internal nodes. Notice that for each 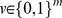 we may define 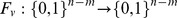 by 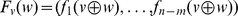 (we suppress the notation for the standard inclusion of the direct sum). Thus we have defined a family of 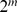 Boolean networks consisting of the internal nodes, one for each vector in 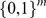.

Suppose that to each external input 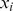 we associate a function 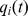 taking values in 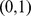 with 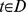, some arbitrary domain representing time. Call 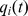 a control function. We suppose that this probability represents the qualitative activation of the species represented by 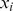 at time 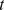. Let 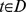 be fixed and consider the probability distribution 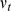 on 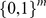 given by 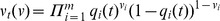. Using this construct 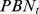, a probabilistic Boolean network where the probability that 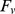 is chosen to update the network is 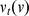. Abusing notation so that 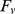 also stands for the state transition matrix of the associated Boolean network, the state transition matrix of the corresponding Markov chain is 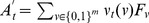. It is important to point out that we may not assume that this Markov chain is irreducible, as we are not considering any arbitrary perturbations of nodes. However, within the state space 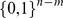 there are recurrent communicating classes of states, and any random walk corresponding to this Markov chain will arrive at one of these sets.

Suppose then that 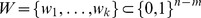 is a recurrent communicating class. Restricting the state transition matrix to W, let 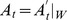. 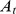 is an irreducible Markov chain. As we have no guarantee that the resulting transition matrix is aperiodic we consider 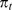, the stationary distribution on 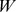 associated to the Markov chain 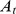. We define the *activity profile* of the W at time 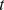, a recurrent communicating class of the original Markov chain, by 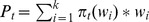. We then interpret the 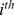 entry of 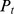 as the qualitative activation of the species represented by node 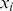 at time 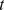. It is important to note that the recurrent communicating classes of 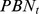 are the same for all times 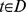. (Thus, as above, we do not need to index W by 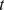.) This can be seen by understanding that the recurrent communicating classes are determined by the semigroup generated by the maps 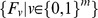, and not by the probabilities associated to each 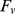 [49]. This is why we take care to assume that each 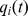 takes values in 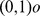 since if at some time 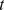, 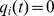 or 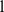, then the semigroup associated to the Markov chain has changed and thus the recurrent communicating classes at that moment may be different. We will refer to this infinite family of PBNs, 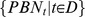, associated to the semigroup 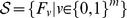 as a *probabilistic Boolean control network*.

Calculating 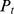 is aided by the fact that it can computed in two steps. First we consider each 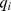 as a formal variable instead as a function of 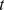. The matrix 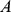 is still stochastic, but its entries are now 0’s, 1’s or polynomials in 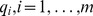. As such application of the Perron-Frobenious theorem allows us to compute the stationary distribution for this irreducible Markov chain as a function which is continuous for all 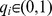. We call these the activity functions for each ergodic set. We then compose these functions with the control function for each 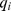 rendering the stationary distribution a function of 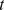. Thus we have continuous functions of 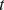 that give activity profile for the ergodic set at time 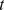. This procedure is demonstrated in a smaller example in Figure 6. We used GAP (Groups, Algorithms, Programming) along with the package Monoid written by James Mitchell in order to compute the recurrent communicating classes for each PBCN. Maple 15 was used to compute the associated stationary distributions. For further mathematical details see [49] and [50].

### Model Construction via The Cell Collective

The Cell Collective (www.thecellcollective.org; [48]), is a collaborative modeling platform for large-scale biological systems. The platform allows users to construct and simulate large-scale computational models of various biological processes based on qualitative interaction information. The platform’s Bio-Logic Builder was used to create this yeast cell cycle models truth tables by specifying the biological qualitative data (adopted from [12]). The Cell Collective’s Knowledge Base component was also used to catalog and annotate all biochemical/biological information for the yeast cell cycle.
